# The 2025 EQUAL *Pneumocystis* Score—an ECMM tool to measure QUALity in *Pneumocystis* pneumonia management

**DOI:** 10.1093/jacamr/dlaf165

**Published:** 2025-09-29

**Authors:** Luise Haensel, Rosanne Sprute, Jan Grothe, Florence Robert-Gangneux, Jean-Pierre Gangneux, Jacques F Meis, Oliver A Cornely, Philipp Koehler

**Affiliations:** Faculty of Medicine and University Hospital Cologne, Department I of Internal Medicine, Excellence Center for Medical Mycology (ECMM), University of Cologne, Herderstrasse 52, Cologne, NRW 50931, Germany; Faculty of Medicine and University Hospital Cologne, Institute of Translational Research, Cologne Excellence Cluster on Cellular Stress Responses in Aging-Associated Diseases (CECAD), University of Cologne, Herderstrasse 52, Cologne, NRW 50931, Germany; Faculty of Medicine and University Hospital Cologne, Department I of Internal Medicine, Excellence Center for Medical Mycology (ECMM), University of Cologne, Herderstrasse 52, Cologne, NRW 50931, Germany; Faculty of Medicine and University Hospital Cologne, Institute of Translational Research, Cologne Excellence Cluster on Cellular Stress Responses in Aging-Associated Diseases (CECAD), University of Cologne, Herderstrasse 52, Cologne, NRW 50931, Germany; German Centre for Infection Research (DZIF), Partner Site Bonn-Cologne, Cologne, NRW, Germany; Faculty of Medicine and University Hospital Cologne, Department I of Internal Medicine, Excellence Center for Medical Mycology (ECMM), University of Cologne, Herderstrasse 52, Cologne, NRW 50931, Germany; Faculty of Medicine and University Hospital Cologne, Institute of Translational Research, Cologne Excellence Cluster on Cellular Stress Responses in Aging-Associated Diseases (CECAD), University of Cologne, Herderstrasse 52, Cologne, NRW 50931, Germany; Mycology Department, Excellence Center for Medical Mycology (ECMM) and French National Center for Chronic Aspergillosis, Rennes University Hospital, Rennes, France; Mycology Department, Excellence Center for Medical Mycology (ECMM) and French National Center for Chronic Aspergillosis, Rennes University Hospital, Rennes, France; CHU Rennes, Inserm, EHESP, IRSET (Institut de Recherche en Santé, Environnement et Travail), UMR_S 1085, Univ Rennes, Rennes, France; Faculty of Medicine and University Hospital Cologne, Department I of Internal Medicine, Excellence Center for Medical Mycology (ECMM), University of Cologne, Herderstrasse 52, Cologne, NRW 50931, Germany; Faculty of Medicine and University Hospital Cologne, Institute of Translational Research, Cologne Excellence Cluster on Cellular Stress Responses in Aging-Associated Diseases (CECAD), University of Cologne, Herderstrasse 52, Cologne, NRW 50931, Germany; Department of Medical Microbiology and Infectious Diseases, Centre of Expertise in Mycology Radboudumc/Canisius Wilhelmina Hospital, Nijmegen, The Netherlands; Faculty of Medicine and University Hospital Cologne, Department I of Internal Medicine, Excellence Center for Medical Mycology (ECMM), University of Cologne, Herderstrasse 52, Cologne, NRW 50931, Germany; Faculty of Medicine and University Hospital Cologne, Institute of Translational Research, Cologne Excellence Cluster on Cellular Stress Responses in Aging-Associated Diseases (CECAD), University of Cologne, Herderstrasse 52, Cologne, NRW 50931, Germany; German Centre for Infection Research (DZIF), Partner Site Bonn-Cologne, Cologne, NRW, Germany; Faculty of Medicine and University Hospital Cologne, Clinical Trials Centre Cologne (ZKS Köln), University of Cologne, Cologne, NRW, Germany; Faculty of Medicine and University Hospital Cologne, Center for Molecular Medicine Cologne (CMMC), University of Cologne, Cologne, Germany; Faculty of Medicine and University Hospital Cologne, Department I of Internal Medicine, Excellence Center for Medical Mycology (ECMM), University of Cologne, Herderstrasse 52, Cologne, NRW 50931, Germany; Faculty of Medicine and University Hospital Cologne, Institute of Translational Research, Cologne Excellence Cluster on Cellular Stress Responses in Aging-Associated Diseases (CECAD), University of Cologne, Herderstrasse 52, Cologne, NRW 50931, Germany; Faculty of Medicine and University Hospital Cologne, Department I of Internal Medicine, Division of Clinical Immunology, University of Cologne, Herderstrasse 52, Cologne, NRW 50931, Germany

## Abstract

**Background:**

*Pneumocystis* pneumonia (PCP) is an opportunistic fungal infection. Several guidelines have been published to support its clinical management. However, the complexity and breadth of these guidelines pose challenges for consistent implementation in routine clinical practice.

**Objectives:**

To develop a scoring tool and quality indicator that facilitates clinical decision making and quantifies adherence to best-practice guidelines for PCP in patients with and without HIV.

**Methods:**

Key recommendations for the diagnosis, treatment and follow-up of PCP were extracted from current guidelines of the European Council on Infections in Leukaemia (ECIL), the American Society of Transplantation Infectious Diseases Community of Practice (AST-IDCOP), and the IDSA guidelines. Each recommendation was assigned a point value ranging from −1 to 3, weighted according to the strength of recommendation and level of evidence.

**Results:**

The proposed 2025 European Confederation of Medical Mycology (ECMM) QUALity (EQUAL) *Pneumocystis* Score consists of 24 items for patients with HIV and 22 for patients without HIV. For diagnosis, bronchoalveolar lavage and immunofluorescence assays received the highest scores due to their superior sensitivity and specificity for sampling and analysis. Trimethoprim/sulfamethoxazole is the treatment of choice and was awarded with the highest score. The addition of corticosteroids for patients with HIV and respiratory failure completes the treatment category. The score is completed with the patient follow-up. For HIV patients a maximum score of 31 and for non-HIV patients a maximum score of 27 is achievable.

**Conclusions:**

The 2025 EQUAL *Pneumocystis* Score offers a concise, evidence-based tool for the optimal management of PCP. This can be useful in daily practice for a quick overview and may be used to measure guideline adherence in research settings. It has not yet been assessed whether higher EQUAL *Pneumocystis* Scores are associated with improved patient outcomes.

## Introduction

*Pneumocystis* pneumonia (PCP) is a respiratory infection caused by the fungal pathogen *Pneumocystis jirovecii*. Patients at risk are individuals with compromised immunity, including those with HIV infection. In the USA, the prevalence of PCP among individuals with HIV was 6.7% in 2002, and this declined to 3.5% in 2014.^1^ This development is due to effective implementation of ART and PCP prophylaxis.^2–4^

The overall incidence of PCP is rising despite the drop in HIV-related cases, due to PCP emerging among new risk populations.^2,3,5–7^ These populations include patients with malignancies, immune-mediated inflammatory diseases or after solid organ transplantation (SOT), particularly those treated with multiple immunomodulating drugs and/or glucocorticoids for more than 3 months.^2,3,5,6^ The underlying immunodeficiency has significant clinical implications for the diagnosis and management of PCP. Patients infected with HIV present with greater numbers of *Pneumocystis* asci whereas inflammatory cell recruitment to the alveoli is limited. In patients without HIV infection, there is rapid respiratory failure with many inflammatory cells detected in bronchoalveolar lavage fluid (BALF) and fewer asci present.^8,9^ The PCP-attributed mortality among non-HIV patients is estimated at 21.9%–22.8% compared with 3.1%–9.9% among patients living with HIV.^1,10,11^

These differences between PCP in HIV and non-HIV patients are reflected in their clinical management. Guidelines from three different societies are providing up-to-date recommendations tailored to specific populations at risk: the European Council on Infections in Leukaemia (ECIL) has published guidelines on the prophylaxis, treatment and diagnosis of PCP in patients with haematological disease.^12–14^ The American Society of Transplantation—Infectious Diseases Community of Practice (AST-IDCOP) issued a guideline covering prevention, management and diagnosis^15^ for SOT recipients. Recommendations for patients living with HIV are available through the quarterly reviewed guidelines from the IDSA.^16^

Diagnosing PCP in clinical practice can be challenging. The clinical presentation with the triad of cough, fever and dyspnoea is non-specific. The definitive diagnosis of PCP by microscopy depends heavily on sample quality and the expertise of the laboratory staff, leading to a high specificity in experienced personnel but limited sensitivity, depending on fungal burden. PCR-based methods, on the other hand, have excellent sensitivity, but positive PCR results may also result from colonization with *P. jirovecii*, complicating the interpretation of positive PCR results.^17^ Currently existing and already published European Confederation of Medical Mycology (ECMM) tools to measure QUALity (EQUAL) in fungal infections aid clinicians with diagnosis, treatment and follow-up of *Aspergillus*, *Candida*, Mucorales, *Cryptococcus*, *Scedosporium*/*Lomentospora* and *Trichosporon* infections as well as chronic pulmonary aspergillosis.^18–26^ They have been translated into several languages and are readily accessible at https://www.ecmm.info/equal-scores/. An accurate and user-friendly EQUAL *Pneumocystis* Score that summarizes guideline recommendations may support treating physicians in well-informed decision making and self-auditing.

We reviewed the four clinical guidelines from ECIL, AST-IDCOP and IDSA and developed a guideline-based score that highlights the strongest recommendations for both HIV-positive and non-HIV patients who develop PCP. This will facilitate clinical management by providing an overview of key recommendations and may be used in future clinical trials to assess guideline adherence with respect to patient outcomes.

## Methods

We conducted a PubMed search to identify guidelines in the English language regarding diagnosis and treatment of PCP. The ECIL, the AST-IDCOP and the IDSA guidelines were chosen as they represent the three major patient groups at risk for PCP. All chosen guidelines are based on a (modified) GRADE approach.

Two independent reviewers extracted key recommendations from the ECIL diagnostic and ECIL treatment guidelines, and from the AST-IDCOP and the IDSA guidelines.^12,14–16^ We organized the recommendations into four groups: clinical suspicion, diagnostic modalities, treatment and follow-up. First, we focused on integrating recommendations of the highest quality of evidence (QoE), namely the ‘AI’ and ‘AII’ recommendations. Next, we added items with lower strength of recommendation (SoR) and quality of evidence. In the final step, we assigned points based on the SoR and QoE of each recommendation to develop the EQUAL *Pneumocystis* Score following the EQUAL score methodology for validated aspergillosis, candidaemia and mucormycosis scores in group consensus.^18–22^ To maintain simplicity, we deliberately excluded recommendations of epidemiological relevance such as genotyping for outbreak investigation or those recommendations not applicable to the majority of patient groups, e.g. specific recommendations for pregnancy.

## Results

### Diagnosis

The diagnosis of PCP requires a combination of clinical, radiological and microbiological features. The evaluated guidelines recommend suspecting PCP in patients with relevant risk factors, a characteristic clinical presentation and chest imaging with suspicious infiltrates.^14,16^ The EQUAL *Pneumocystis* Score 2025 assigns three points for evaluating the clinical signs and underlying risk factors, as well as initiating timely diagnostic and therapeutic measures (Table 1). Several patient groups are at risk of developing PCP. Patients with HIV are at increased risk if ART-naive and ART non-adherent and showing a CD4 cell count <200 cells/mm^3^.^16^ Risk factors in patients without HIV can be variable and the actual hazard may vary. Current data show that patients at high risk of PCP are haematopoietic stem cell transplant recipients (particularly >100 days post-transplant) and lung or kidney transplant recipients.^13,16^ Patients at moderate risk are patients with malignancies or autoimmune diseases receiving a combination of immunosuppressive therapy including glucocorticoids with a median dose of 30 mg prednisone equivalent daily for at least 2–3 months.^38^ Patients treated with a single immunomodulating drug are at low risk.^13,17,38^ An exception are patients treated with B cell–depleting agents, who seem to be at moderate to high risk.^39^

**Table 1.** The 2025 EQUAL *Pneumocystis* Score derived from available guidelines^a^

Quality indicator	Strength and level of recommendation^b^				Comment	Score	
	ECIL diagnosis guideline 2016^14^	ECIL treatment guideline 2016^12^	AST-IDCOP guideline 2019^15^	NIH-HIVMA-IDSA guideline 2025^16^		HIV patients	non-HIV patients
Clinical suspicion							
Prompt diagnostic procedures when clinical criteria are matching	—	A-III	—	—	Risk factor plus triad of fever, dyspnoea and hypoxia plus radiographic signs of PCP^17^	3	3
Diagnostic modalities							
Respiratory tract specimen							
BALF **OR**	A-II	—	Strong/high	—	Brown *et al.* reports qPCR sensitivity 98.7%, specificity 89.2%; cPCR sensitivity 97.2%, specificity 95.4%. Other comparative analyses are not available^14–16,27,28^	3	3
Transbronchial biopsy **OR**	—	—	Strong/moderate	—	Limited use due to possible complications and limited data (bleeding, lung injury)	2	2
Open-lung biopsy **OR**	—	—	Strong/low	—	According to literature standard procedure for diagnosis, usually not required; limited recommendation due to possible complications (bleeding, lung injury) and limited data^15^	1	1
Induced sputum **OR**	B-II	—	Strong/high		Requires inhalation of NaCl for at least 20 min before sampling.^29^ qPCR sensitivity 98%, specificity 81%; cPCR sensitivity 95.6%, specificity 91.7%^27^	2	2
Non-invasive specimens (nose swabs, non-induced sputum)		—	Strong/low	—	Insufficient alternatives, low organism burden. Only recommended when BALF, biopsy or induced sputum are not available Brown *et al.*: qPCR sensitivity 89.2%, qPCR specificity 90.5%, cPCR sensitivity 78.7%, cPCR specificity 96%^15,27^	1	1
Diagnostic modality							
Immunofluorescence including calcofluor white **OR**	A-II	—	Strong/high	—	Sensitivity 48%–100%; specificity 82%–100%^14,15,30,31^	3	3
Other staining	—	—	Strong/high		If IF is unavailable, a combination of calcofluor white (CW) or Gömöri methenamine silver (GMS) plus Giemsa or toluidine blue (TBO) is recommended. Among these, calcofluor white or Gömöri methenamine silver are the most predictive.^15^ Sensitivity CW: 57%–78%, specificity: 99.6%. Sensitivity GMS: 31%–97%, specificity: 92%–100%. Sensitivity TBO: 49%–94%, specificity: 96.8%–100%^30–32^	2	2
Real time quantitative PCR	A-III	—	Strong/low	—	NPV is 100%, essential to rule out PCP. In case of a positive result, until now insufficient discrimination between colonization and infection; recent studies try to establish a standardized cut-off^33^	3	3
Blood testing							
Serum β-D-glucan	A-II	—	Weak/moderate	—	Recently evaluated by a meta-analysis by Prosty *et al.*^34^	2	2
Lactate dehydrogenase (LDH)	—	—	Weak/low	—	Unspecific, usually positive in PCP^14,15^	1	1
Treatment							
Drug							
SXT (15–20 mg/kg/75–100 mg/kg) **OR**	—	A-IIr	Strong/Moderate	AI	Current standard therapy^12,15,16^	3	3
Alternatives: pentamidine (4 mg/kg/day), primaquine/clindamycin (30 mg/day + 600 mg every 8 h), atovaquone (750 mg every 8–12 h daily), dapsone 100 mg plus 15 mg/kg/day	—	C-II	Strong/moderate-low	B-II	Switch from SXT to alternatives should be avoided if adverse events are manageable. Evidence is scarce^12,15,16^	1	1
Route of administration							
Mild symptoms: oral administration **OR**	—	B-IIt	—	AI	Only if no gastrointestinal impairment is present^12,16^	2	2
Moderate to severe symptoms: IV administration	—	A-IIu	—	AI	Current standard therapy^12,16^	2	2
Duration							
Standard duration of 3 weeks	—	B-IIt	—	—	Current standard therapy^12^	2	2
Use of corticosteroids							
HIV-negative: no use of glucocorticoids	—	B-IIh	Strong/Low	—	In case of ARDS decision is to be made on an individual basis^12,15^	—	—
HIV-positive: adjunctive corticosteroids in moderate to severe cases	—	A-I	—	—	Gold standard^16^	2	—
Follow-up							
β-D-glucan for follow-up		D-II	—	—	β-D-glucan only correlates weakly with treatment outcome^35^	−1	−1
PCR from BALF for follow-up	A-II				*Pneumocystis* DNA can persist even after successful treatment^36^	−1	−1
CT scan	—	A-III	—	—	Decrease of pulmonary infiltrates can be observed after a median of 13 days^37^	2	2
Secondary prophylaxis	—	A-IIh	—	AI	Can be stopped in patients with HIV, if CD4 cell counts have increased to ≥200 cells/mm^3^ for ≥3 months after initiation of ART (AII). Duration in patients without HIV remains unknown^16^	3	3
Only HIV-positive: ART initiated within 2 weeks of diagnosis of PCP	—	—	—	AI	Peculiarities in intubated patients, consider gastroenteric impairment and potential drug–drug interactions^16^	2	—
**Maximum score**						**31**	**27**

ARDS, acute respiratory distress syndrome; AST-IDCOP, American Society of Transplantation—Infectious Diseases Community of Practice; BALF, bronchoalveolar lavage fluid; CD, cluster of differentiation; cPCR, conventional PCR; CW, calcofluor white; ECIL, European Council on Infections in Leukaemia; GMS, Gömöri methenamine silver; HIVMA, HIV Medicine Association; IF, immunofluorescence; NPV, negative predictive value; PCP, *Pneumocystis* pneumonia; PJP, *Pneumocystis jirovecii* pneumonia; qPCR, quantitative PCR; SXT, trimethoprim/sulfamethoxazole; TBO, toluidine blue.

Sources: ECIL diagnosis guideline, ECIL guidelines for the diagnosis of PJP in patients with haematological malignancies and stem cell transplant recipients (2016); ECIL treatment guideline, ECIL guidelines for treatment of PJP in non–HIV-infected haematology patients (2016); AST-IDCOP guideline, guidelines from the American Society of Transplantation Infectious Diseases Community of Practice; NIH-HIVMA-IDSA guideline, Panel on Guidelines for the Prevention and Treatment of Opportunistic Infections in Adults and Adolescents With HIV. Guidelines for the Prevention and Treatment of Opportunistic Infections in Adults and Adolescents With HIV. NIH HIVMA and IDSA. Available at https://clinicalinfo.hiv.gov/en/guidelines/adult-and-adolescent-opportunistic-infection.

^b^Grade of recommendation: A, the guideline group strongly supports a recommendation for use; B, the guideline group moderately supports a recommendation for use; C, the guideline group marginally supports a recommendation for use; D the guideline group supports a recommendation against use. Level of evidence: I, evidence from at least one properly designed randomized, controlled trial; II, evidence from at least one well-designed clinical trial, without randomization; from cohort or case-controlled analytic studies (preferably from more than one centre); from multiple time series; or from dramatic results of uncontrolled experiments; III, evidence from opinions of respected authorities, based on clinical experience, descriptive case studies, or reports of expert committees. Added index: t, transferred evidence, i.e. results from different patients’ cohorts, or similar immune-status situation; h, comparator group: historical control; u, uncontrolled trials.

Besides the symptom triad of dyspnoea, fever and dry cough, additional tachypnoea and hypoxia contribute to the suspicion of PCP.^14–16^ Patients infected with HIV present the symptom triad regularly, whereas non–HIV-infected patients show less specific symptoms, with dyspnoea and respiratory failure being more common.^40,41^

Radiographic imaging shows characteristic features, with bilateral symmetric reticular opacities in chest X-rays, and ground glass opacities with peripheral sparing, thickened interlobular septae, and the crazy paving phenomenon with CT.

Diagnostic tests for PCP can be performed on different respiratory tract samples.^27^ BALF is the sample with the highest sensitivity and specificity [e.g. conventional PCR (cPCR) sensitivity 97.2%, specificity 95.4%^27^] for both HIV and non-HIV populations. Induced sputum (IS) has comparable test performance for individuals infected with HIV (e.g. cPCR sensitivity 95.6%, specificity 91.7%^27^). In contrast, for non-HIV patients the results are less consistent. Two meta-analyses (Brown *et al.*^27^ and Senécal *et al.*^42^) evaluated PCR diagnostics in BALF and IS. Brown *et al.* did not report relevant differences for cPCR and quantitative PCR (qPCR) performed on these samples, whereas Senécal *et al.* found reduced specificity, particularly in IS. We therefore assigned three points for using BALF and two points for IS. Nasopharyngeal swabs or expectorated sputum have low sensitivity and therefore should only be used when no other options are feasible.^14^ The AST-IDCOP also mentions histopathology via transbronchial biopsy or video-assisted thoracoscopy in SOT recipients, which is generally not required.^15^ We allocated two points to this procedure as an alternative diagnostic strategy due to its strong recommendation supported with moderate evidence. The AST-IDCOP includes open-lung biopsy in the diagnostic modalities. We assigned one point, as we found little supporting evidence and possible risks of complications such as bleeding and lung injury.

The standard microbiological test to detect the pathogen can be achieved via visualization of asci and trophic forms of *P. jirovecii* by immunofluorescence (IF). We valued the procedures with three points. Direct staining of BALF with May–Grünwald, Wright–Giemsa or Grocott–Gömöri methenamine silver stain is less sensitive and these techniques are therefore awarded two points.^14–16^ It is important to note that negative microscopy does not rule out PCP, especially in patients without HIV, because they appear to have fewer asci and trophic forms present in the lung.^14^

Recently, more data have become available on the use of PCR techniques demonstrating a high sensitivity and a negative predictive value (NPV) of 100%.^42–44^ As a result we allocated three points for this. Higher fungal loads are associated with low cycle threshold (ct) values, but until now no validated cut-off values have been determined to differentiate infection from colonization.

Another indicator suggestive of PCP is an elevated lactate dehydrogenase (LDH). We therefore allocated one point for the laboratory testing of LDH. Alongside LDH, elevated β-D-glucan (BDG) in serum also supports suspicion for PCP infections.^14^ Hence, BDG measurement was weighted with two points. However, it is important to emphasize that although both markers may aid in the diagnostic process, interpretation of BDG results has to occur in the context of the patient’s comedications and potential coinfections.^16^ Furthermore, in non-HIV patients, data remain sparse and it is still unclear whether a negative BDG reliably rules out PCP.^45^

### Treatment

The first-line treatment for PCP is trimethoprim/sulfamethoxazole, also known as co-trim, septrin or bactrim, administered at a dosage of trimethoprim 15–20 mg/kg and sulfamethoxazole 75–100 mg/kg. An oral administration route is appropriate in mild and moderate cases without factors impairing gastrointestinal absorption. In moderate to severe cases, IV application is recommended and an oral administration can be considered once the patient’s clinical condition is improving. Treatment duration should be at least 14 days in moderate cases and 21 days in severe cases.^12,16^ The disease severity, categorized as mild, moderate or severe, is outlined in Table 2. We assigned three points for the choice of medication, two points for the correct administration, and granted another two points for the correct treatment duration. For alternative treatments, such as pentamidine (4 mg/kg/day), primaquine/clindamycin (30 mg/day + 600 mg every 8 h), atovaquone (750 mg every 8–12 h daily) and dapsone (100 mg plus 15 mg/kg/day), we allocated two points. Prior to switching to second-line treatments, a thorough evaluation should determine whether the adverse events of trimethoprim/sulfamethoxazole are manageable, as evidence supporting alternative regimes is limited.^16^ Adverse effects of trimethoprim/sulfamethoxazole are frequent (20%–80%) and include rashes (rarely Stevens–Johnson syndrome), fever, leukopenia, thrombocytopenia, azotaemia and hyperkalaemia. Mild reactions can be managed symptomatically with antihistamines, antiemetics and antipyretics. In case of severe reactions or life-threatening events, therapy should be discontinued and switched to alternative therapies.^16^

**Table 2.** Severity grading of PCP according to the current guidelines^12,46^

	Mild	Moderate	Severe
HIV patients	pO_2_ at rest, room air >70 mmHg	pO_2_ at rest, room air <70 mmHg	pO_2_ at rest, room air ≤35 mmHg
Non-HIV patients	pO_2_ at rest, room air 82.5 mmHg	pO_2_ at rest, room air 60.75–82.5 mmHg	pO_2_ at rest, room air <60 mmHg

pO2, partial pressure of oxygen.

We assigned one point for the use of corticosteroids in individuals with HIV. In this population, the use of adjunctive corticosteroids has been shown to improve the overall mortality in cases with moderate to severe PCP.^47^ Prednisone should be initiated as soon as possible and tapered according to current guideline recommendations.^16^

In contrast, adjunctive corticosteroids are not routinely recommended in PCP patients without HIV infection. However, in case of respiratory failure they might be considered on an individual basis.^15^ Given the lack of guideline recommendations and the limited quality of available data, we chose not to include corticosteroid use in non–HIV-infected individuals in our scoring system.

### Follow-up

Vogel *et al.*^37^ reported radiographic improvement becoming evident after a median of 13 days. Currently, no other systematic study, especially including patients with HIV, has investigated this matter to our knowledge. The ECIL supports this with a A-III recomendation, and case reports^48,49^ qualitatively align with the findings of Vogel *et al*. We assigned two points for performing follow-up imaging. Monitoring therapy success using BDG or PCR-based DNA detection is not recommended as both can be elevated after successful treatment.^12,35,36^ Therefore, we deducted one point each for the use of serum BDG measurement or PCR from BALF for follow-up purposes.

Secondary prophylaxis is encouraged for all patients in the EQUAL score and weighted with three points. In individuals with HIV, prophylaxis should be initiated immediately after completing PCP treatment and continued until the CD4 count exceeds 200 cells/mm³ for at least 3 months. In patients with a CD4 count between 100 and 200 cells/mm³ and undetectable viral RNA for 3–6 months, prophylaxis can also be discontinued. Regardless of viral load, secondary prophylaxis should be restarted if the CD4 count falls under 100 cells/mm³ or if it remains between 100 and 200 cells/mm³ with detectable viral RNA.^16^

In non-HIV patients, secondary prophylaxis is also recommended after successful treatment of PCP. However, the recommendation on the duration remains vague. The ECIL guideline advises deciding on an individual basis once the immune system of the patients has fully recovered. Although secondary prophylaxis is not recommended in SOT recipients,^15^ treatment similarities regarding treatment regimens and the use of immunomodulating drugs with haemato-oncological patients may also support the use of secondary prophylaxis.

### ART and immune reconstitution inflammatory syndrome

We also incorporated recommendations on ART. Although for a long time immune reconstitution inflammatory syndrome (IRIS) was feared as a complication in early ART initiation, newer studies revealed that early initiation of ART was non-inferior or superior to delayed initiation with regards to outcome.^50–52^ Therefore, ART should ideally be started within 2 weeks of diagnosing PCP.^16^ We awarded initiation of ART within 2 weeks of diagnosing PCP with three points.

Maximum points achievable per category are summarized in Table 3.

**Table 3.** The 2025 EQUAL *Pneumocystis* Score^a^

	HIV patients	Non-HIV patients
Clinical suspicion	3	3
Diagnosis	12	12
Treatment	9	7
Follow-up	7	5
Maximum score	31	27

^a^Maximum points achievable per category.

## Discussion

In 2018, the European Confederation of Medical Mycology (ECMM) introduced the first EQUAL score to quantify adherence to clinical guidelines in candidiasis.^53^ Since then, EQUAL scores have been developed for various other fungal infections, including invasive and chronic pulmonary aspergillosis, cryptococcosis, mucormycosis, trichosporonosis and scedosporiosis/lomentosporiosis.^20–23,26,54^ These scoring tools have also been used in several studies to investigate the relationship between guideline adherence and patient outcome.^18,19,53,55–59^ With the EQUAL *Pneumocystis* Score, our aim was to develop a user-friendly, reasonable and scientifically robust scoring system.

Although all included items are supported by current guidelines, debate remains due to lack of sufficient original data. This is especially relevant in the non-HIV patient population. We did not include radiographic imaging as an independent item, because of its low sensitivity and specificity. Ground glass opacities can be observed in various lung pathologies such as severe acute respiratory syndrome coronavirus 2 (SARS-CoV2), pulmonary oedema or drug toxicities.^60^ Even though we assigned three points for the qPCR analysis, the procedure fails to distinguish colonization from true infection. As of today, no standardized ct values or gene loci have been defined for optimized diagnostic accuracy.^33,61,62^ Despite the promising results that Senécal *et al.*^42^ presented on nasopharyngeal swaps, oral washes and serum tests, for development of the EQUAL score we followed current guideline recommendations on non-invasive specimens.

Furthermore, the optimal application of BDG remains unclear. We assigned two points for BDG measurement because it can add valuable information in the diagnostic process. Recent meta-analyses are questioning this recommendation for patients without HIV as BDG levels tend to be higher in patients with HIV.^45,63^ In addition, BDG cut-off values remain unvalidated in PCP. The results of the two commercially available tests—FUJIFILM Wako and Fungitell^®^—are difficult to compare due to their different measurement techniques.^64,65^

Besides optimizing diagnostic techniques, recent research questioned current treatment approaches. The optimal dosage of trimethoprim/sulfamethoxazole remains a topic of ongoing debate.^66,67^ A prospective randomized trial currently underway (NCT 048510154851015) may help to determine the optimal dosage. Studies investigating the treatment regimens have mostly been conducted in white male patients and individuals infected with HIV. Diverse cohorts have only been studied in non-randomized clinical trials with small sample sizes.^68^

New therapeutic approaches are currently under investigation and may lead to significant changes in management in the coming years. These include ongoing studies on the use of echinocandins including rezafungin and the novel glucan synthase inhibitor ibrexafungerp.^9,69,70^ We did not include the use of adjunctive corticosteroids in patients without HIV according to the ECIL recommendations. However, a prospective randomized controlled trial is underway and may provide further evidence to guide clinical practice (NCT02944045).

We included recommendations on secondary prophylaxis in non-HIV patients, such as those with haematological malignancies, transplantation or prolonged immunosuppression. However, the duration of secondary prophylaxis in particular is still undefined in these populations and further evidence is needed to address the optimal length.

Data on life-threatening IRIS after PCP treatment initiation remain limited, with studies reporting varying incidence rates. IRIS typically occurs several weeks after initiation of ART. Although early initiation of ART within the first 2 weeks is widely recommended—and accordingly considered in our score—this approach may not be suitable in all clinical contexts, e.g. for patients with HIV and cryptococcal meningoencephalitis coinfection who are not on ART. The A5164 trial, which demonstrated improved outcomes with early ART initiation, included mainly patients from the northern hemisphere and the study excluded patients with severe PCP and comorbidity with TB. It is not clear whether those patients also profit from early initiation of ART.^51^

Although several uncertainties remain in the diagnosis and treatment of PCP, we have summarized the most important recommendations from the ECIL, ISDA and AST-IDCOP guidelines and developed the 2025 EQUAL *Pneumocystis* Score. This score is intended as a tool to evaluate adherence to current guidelines. However, it remains uncertain whether adherence to guidelines alone directly influences individual patient outcomes. Importantly, in special clinical situations, e.g. pregnancy, the score may be overly simplistic, and individualized diagnosis, treatment and prophylaxis strategies are required. Validation of the established EQUAL *Pneumocystis* Score in patient cohorts represents an essential next step, as has been performed for previously developed EQUAL scores.^18–20^

The 2025 EQUAL *Pneumocystis* Score may align patient management with current management recommendations and enhance comparability among studies conducted in different centres.

## Transparency declarations

L.H. has nothing to declare. R.S. reports grants from the German Center for Infection Research and the Ministry of Culture and Science of the State of North Rhine-Westphalia, lecture and speaker honoraria from Akademie für Infektionsmedizin e.V., Ärztliche Akademie für medizinische Fort- und Weiterbildung in Nordrhein, FomF GmbH, Infektio Saar Netz, Hikma, Mundipharma and Pfizer, and travel support from the ECMM, ESCMID, ISHAM, Page Medical and Pfizer; all outside of the submitted work. J.G. has nothing to declare. J.-P.G. has nothing to declare. F.R.-G. has nothing to declare. J.F.M. has nothing to declare. O.A.C. reports grants or contracts from iMi, iHi, DFG, BMBF, Cidara, DZIF, EU-DG RTD, F2G, Gilead, MedPace, MSD, Mundipharma, Octapharma, Pfizer, Scynexis; consulting fees from Abbvie, AiCuris, Basilea, Biocon, Boston Strategic Partners, Cidara, Elion Therapeutics, Gilead, GSK, IQVIA, Janssen, Matinas, MedPace, Menarini, Melinta, Molecular Partners, MSG-ERC, Mundipharma, Noxxon, Octapharma, Pardes, Pfizer, PSI, Scynexis, Seres, Seqirus, Shionogi, The Prime Meridian Group; speaker and lecture honoraria from Abbott, Abbvie, Akademie für Infektionsmedizin, Al-Jazeera Pharmaceuticals/Hikma, amedes, AstraZeneca, Deutscher Ärzteverlag, Gilead, GSK, Grupo Biotoscana/United Medical/Knight, InfectoPharm, Ipsen Pharma, Medscape/WebMD, MedUpdate, MSD, Moderna, Mundipharma, Noscendo, Paul-Martini-Stiftung, Pfizer, Sandoz, Seqirus, Shionogi, streamedup!, Touch Independent, Vitis; payment for expert testimony from Cidara; participation on a DRC, DSMB, DMC, Advisory Board for AstraZeneca, Cidara, IQVIA, Janssen, MedPace, Melinta, PSI, Pulmocide, Vedanta Biosciences. P.K. reports grants or contracts from the German Federal Ministry of Research and Education (BMBF) B-FAST (Bundesweites Forschungsnetz Angewandte Surveillance und Testung) and NAPKON (Nationales Pandemie Kohorten Netz, German National Pandemic Cohort Network) of the Network University Medicine (NUM), the State of North Rhine-Westphalia and the Dr Heinz Lux-Stiftung Foundation; consulting fees from Ambu GmbH, Gilead Sciences, infill healthcare communication GmbH, Mundipharma Research Ltd, Noxxon N.V. and Pfizer Pharma; honoraria for lectures from Akademie für Infektionsmedizin e.V., Ambu GmbH, Astellas Pharma, BioRad Laboratories Inc., Datamed GmbH, European Confederation of Medical Mycology, Gilead Sciences, GPR Academy Ruesselsheim, HELIOS Kliniken GmbH, Jazz Pharmaceuticals Germany GmbH, Lahn-Dill-Kliniken GmbH, medupdate GmbH, MedMedia GmbH, MSD Sharp & Dohme GmbH, Pfizer Pharma GmbH, Sanofi-Aventis Deutschland GmbH, Scilink Comunicación Científica SC, streamedup! GmbH, University Hospital and LMU Munich and VITIS GmbH; participation on an Advisory Board from Ambu GmbH, Gilead Sciences, Mundipharma Research Limited and Pfizer Pharma; a filed patent at the German Patent and Trade Mark Office (DE 10 2021 113 007.7); other non-financial interests from Elsevier, Wiley and Taylor & Francis online outside the submitted work.
